# Genetic structure and historical demography of inland wetland fish using the endangered Lisbon arched-mouth nase as a case-study

**DOI:** 10.1038/s41598-025-05280-x

**Published:** 2025-07-02

**Authors:** Giulia Riccioni, Manuel Curto, Carlos D. Santos, Maria Judite Alves, Hugo F. Gante, Filipe Ribeiro, Ana Veríssimo

**Affiliations:** 1 DIANA – Department of Animal Science, Nutrition and food – Faculty of Agriculture, Catholic University of Sacred Heart, Piacenza, Italy; 2 MARE – Marine and Environmental Sciences Center/ARNET – Aquatic Research Network, Faculty of Sciences, University of Lisbon, Lisbon, Portugal; 3 CIBIO, Centro de Investigação em Biodiversidade e Recursos Genéticos, InBIO Laboratório Associado, Campus de Vairão, 4485-661 Vairão, Portugal; 4 BIOPOLIS Program in Genomics, Biodiversity and Land Planning, CIBIO, Campus de Vairão, 4485-661 Vairão, Portugal; 5 MARE – Marine and Environmental Sciences Centre/ARNET – Aquatic Research Network, NOVA School of Science and Technology, NOVA University of Lisbon, Lisbon, Portugal; 6 cE3c – Centre for Ecology, Evolution and Environmental Changes/CHANGE – Global Change and Sustainability Institute, University of Lisbon, Lisbon, Portugal; 7 National Museum of Natural History and Science, University of Lisbon, Lisbon, Portugal; 8 Division Ecology, Evolution and Biodiversity Conservation, Department of Biology, KU Leuven, Charles Deberiotstraat 32, 3000 Leuven, Belgium; 9 Section Vertebrates, Department of Biology, Royal Museum for Central Africa, Leuvensesteenweg 17, 3080 Tervuren, Belgium

**Keywords:** Floodplain habitats, Tidal freshwaters, Freshwater fish, Population structure, Habitat tracking, Biodiversity, Conservation biology, Freshwater ecology, Wetlands ecology, Genetics

## Abstract

Inland wetlands are highly diverse, productive ecosystems at the transition between terrestrial and aquatic environments, providing major services to society. They are also under high human pressure and have suffered a progressive decline in total area globally. Here, we describe the genetic diversity and demography of the endangered Lisbon arched-mouth nase *Iberochondrostoma olisiponense* occurring in the inland wetlands of a major river in southwestern Europe, within a context of extreme habitat changes. Our results highlight the presence of well-structured small population nuclei with evidence of sporadic gene flow at historical and contemporary time scales. Historical reconstructions suggest population isolation consistent with periods of unsuitable habitat conditions severing population connectivity, while small population sizes may be due to limited available habitat coupled to historical as well as recent restricted connectivity among river tributaries. Habitat contraction or loss due to decreased rainfall associated with climate change is expected to impact inland wetland fish regionally, raising additional conservation concerns on species with low genetic diversity and small effective population sizes as *I. olisiponense*. This study may serve as a proxy for taxa occurring in similar inland wetland ecosystems and may inform conservation efforts and planning regarding the expected drivers of population demographics, distribution and connectivity.

## Introduction

Inland wetlands are important transition zones between terrestrial and aquatic environments characterized by high productivity and biodiversity^1^. These include a variety of habitats such as forested and non-forested peatlands, marshes and swamps on alluvial lowlands, among others^2^. Overall, these ecosystems provide important services such as controlling river discharge volumes and speed, improving water quality, controlling shore erosion, providing food and habitat sources, to name just a few^3,4^. Indeed, they are historically important areas for human settlement as they provide access to freshwater, productive soils for agriculture and animal production, as well as means for transporting people and goods via river channels^5,6^. Consequently, natural inland wetlands are currently under intense human-driven pressure globally, which have led to declines in their total area while remaining mostly unprotected^7,8^.

The location and extent of inland wetlands are intimately dependent on river flow and sediment regimes, as well as on the physiographic/topographic features and vegetation cover of the landscape^9^. All these features vary considerably in space (e.g. latitude;^2^) as well as in time (e.g. contemporary vs. geological past). Notably, changes in global sea levels associated with glacial and interglacial cycles have profound impacts on the distribution and extent of inland wetlands^10^ and, therefore, on their living communities. Nevertheless, anthropogenic pressure during the last centuries has contributed to a concerning reduction in extent and/or alteration of wetlands globally^11,12^

Inland wetland communities, particularly those occurring in areas closest to river mouths, are expected to be influenced most strongly by the effects of marine regressions and transgressions, hydrological regime shifts (natural and man-made) and human occupation^13^. These areas are characterized by daily, seasonal and/or permanent flooding and are, thus, highly dynamic in nature^6^. Tidal freshwater wetlands are a particular case within inland wetlands, as transition zones between marine and freshwaters under daily tidal influence^14^. However, few studies have focused on understanding the dynamics, population structure and historical demography of inland wetland aquatic species occurring exclusively in these areas, and particularly of fish (e.g.^15,16^).Towards this end, we focused on a case-study fish species—the Lisbon arched-mouth nase *Iberochondrostoma olisiponense*—and assessed the levels and distribution of genetic diversity throughout its known range.

**Fig. 1:**
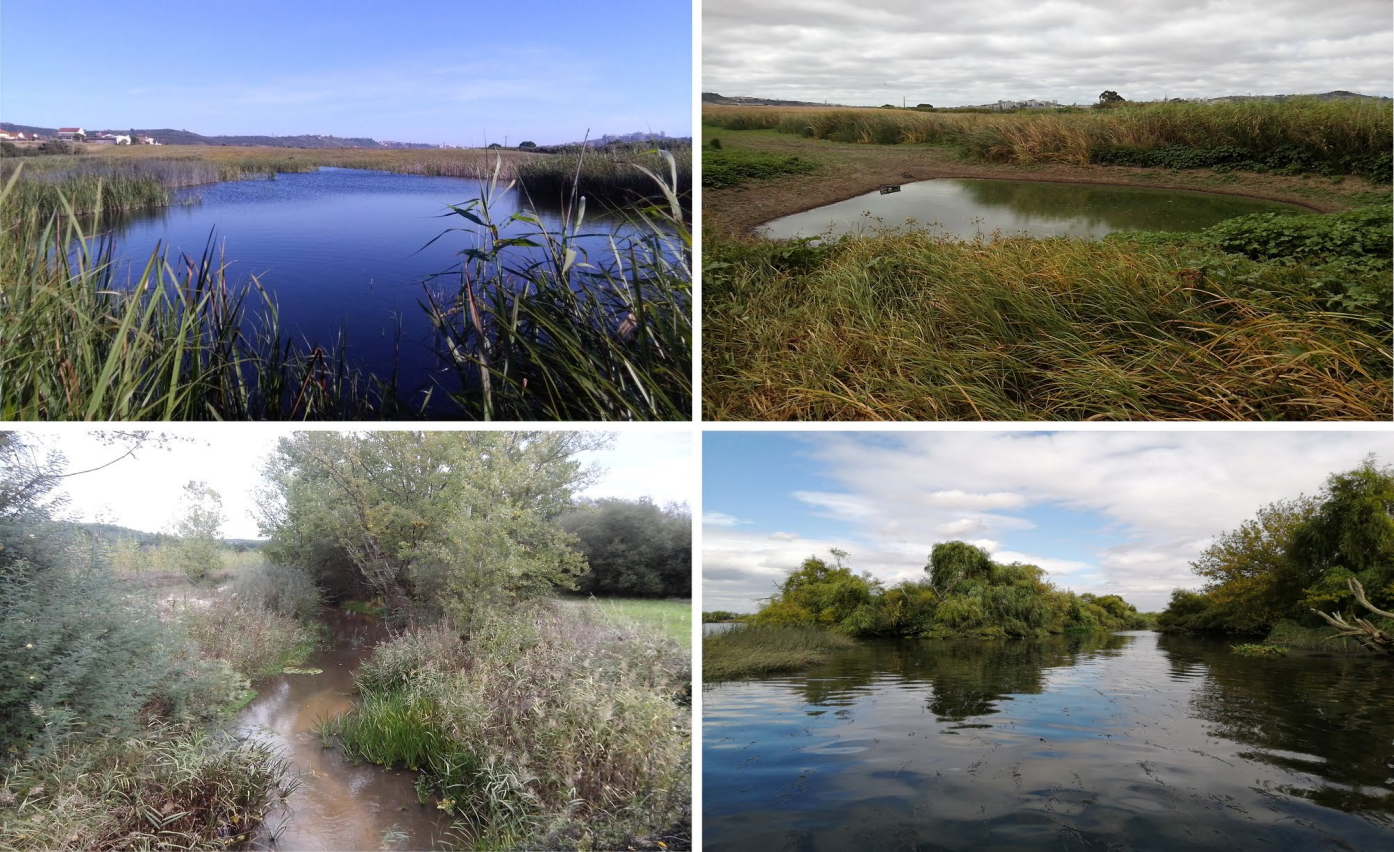
Typical habitat of Lisbon arched-mouth nase *Iberochondrostoma olisiponense*. Top panels: a freshwater marsh at the Trancão basin (38° 51′ 1.64″ N, 9° 8′ 52.78″ W) in winter (left) and summer (right, depicting a naturalized well). Bottom panels: slow-current tributary, Muge River (left panel, 39° 8′ 20.51″ N, 8°31′26.95″ W), and a tidal freshwater side channel on the Tagus mainstream (right panel, 39° 2′ 51.9″ N 8°48′12.7″ W).

**Fig. 2:**
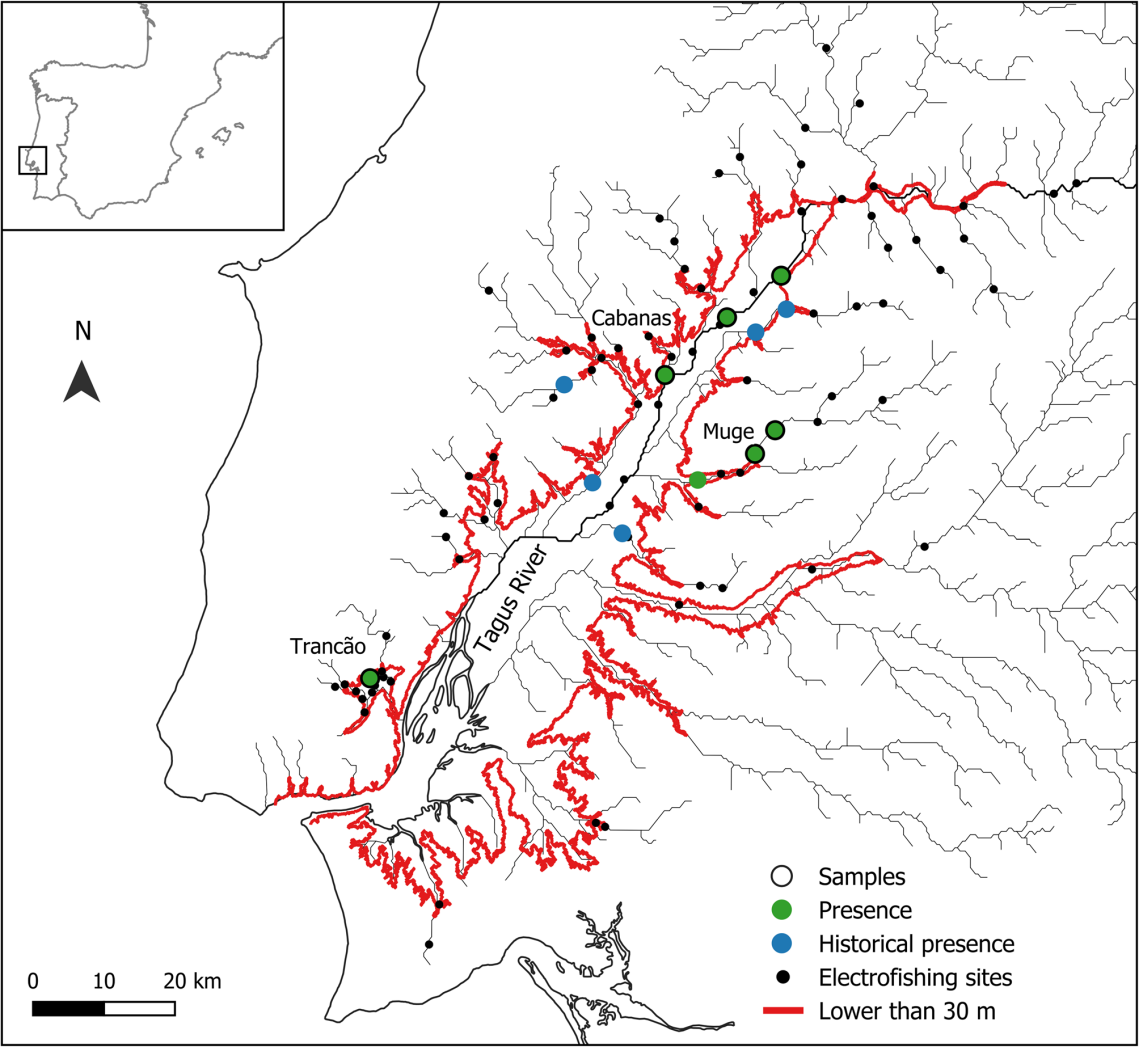
Map of the lower Tagus section, in the western Iberian Peninsula (inset map), showing the 86 sites surveyed with electrofishing (green and black dots) for the Lisbon arched-mouth nase *Iberochondrostoma olisiponense*. Sites where species´ presence was confirmed are highlighted in green, and sites where samples for genetic analysis were obtained are circled in black contour. Sites where species´ presence was not confirmed are highlighted in black, and sites with historical records are highlighted in blue. Most sites with species´ presence (blue and green) are bounded by the 30 m altitude contour delimiting the inland wetlands in the lower Tagus section (sensu^12^).

The Lisbon arched-mouth nase is a small-sized leuciscid fish occurring exclusively in inland wetlands of low altitude (generally ~ 10 m altitude, and rarely above 60 m)^17^, such as marshes and slow-current rivers, including tidal freshwater river sections (^18,19^; Ribeiro F., pers. observation; Figs. 1 and 2). The species is endemic to the lower section of the Tagus River (Portugal), one of the major rivers in the Iberian Peninsula. The Tagus has a length of ~ 1000 km from its origin in eastern Spain to the mouth in Lisbon, western Portugal, with a catchment area of ~ 81 000 km^2^ leading to the largest estuary in Europe^20^. The Lower Tagus Valley is a large alluvial plain bounded by Pleistocene terrace sediments with an extremely low gradient (< 0.24 m/km) and high seasonal flow, and thus susceptible to sea level fluctuations, saline water intrusions and extended periods of freshwater residency^5,19,20^. The Lisbon arched-mouth nase exhibits a narrow range and low occupancy in the lower section of the Tagus, but may be locally abundant^17^. Initially thought to occur only in some tributaries, it was later found to live also in the vegetated, low-current side channels in the Tagus mainstream (Fig. 1)^17^. The Lisbon arched-mouth nase is currently listed as Endangered by the Portuguese Red List Book^18^ due to the current restricted area of occurrence, including only a handful of sites^17,21^, and declining population trends^18^. Thus, this study provides important information on the structure, connectivity and genetic diversity of current population nuclei of the Lisbon arched-mouth nase to inform future conservation plans.

## Results

### Exploratory data analysis

Out of the initial 89 individuals sampled and processed for DArTseq, three individuals from Muge were filtered out due to missing data > 20%. The filtered dataset, including the two *I. lusitanicum* specimens, is composed of 86 genotypes and 8,067 binary SNPs and 0.7% missing data. A neighbor-joining tree illustrates the genetic difference between species, and the higher genomic similarity of *I. olisiponense* across sampling sites except for a single individual from Muge that clusters as basal to the remaining *I. olisiponense* (Supplemental Figure S1). The Bayesian analysis carried out on the filtered dataset using Structure 2.3 revealed the highest delta *K* value for *K* = 3 (Supplemental Figure S2). The coefficient of membership plot identified the two *I. lusitanicum* individuals as belonging to a separate cluster, and showed an admixed ancestry from *I. olisiponense* and *I. lusitanicum* for the same individual from Muge at the base of *I. olisiponense* cluster in the neighbor-joining tree (Supplemental Figure S2 and S3). The putative hybrid individual and the two *I. lusitanicum* were removed from further downstream analyses.

### Genetic diversity

**Fig. 3:**
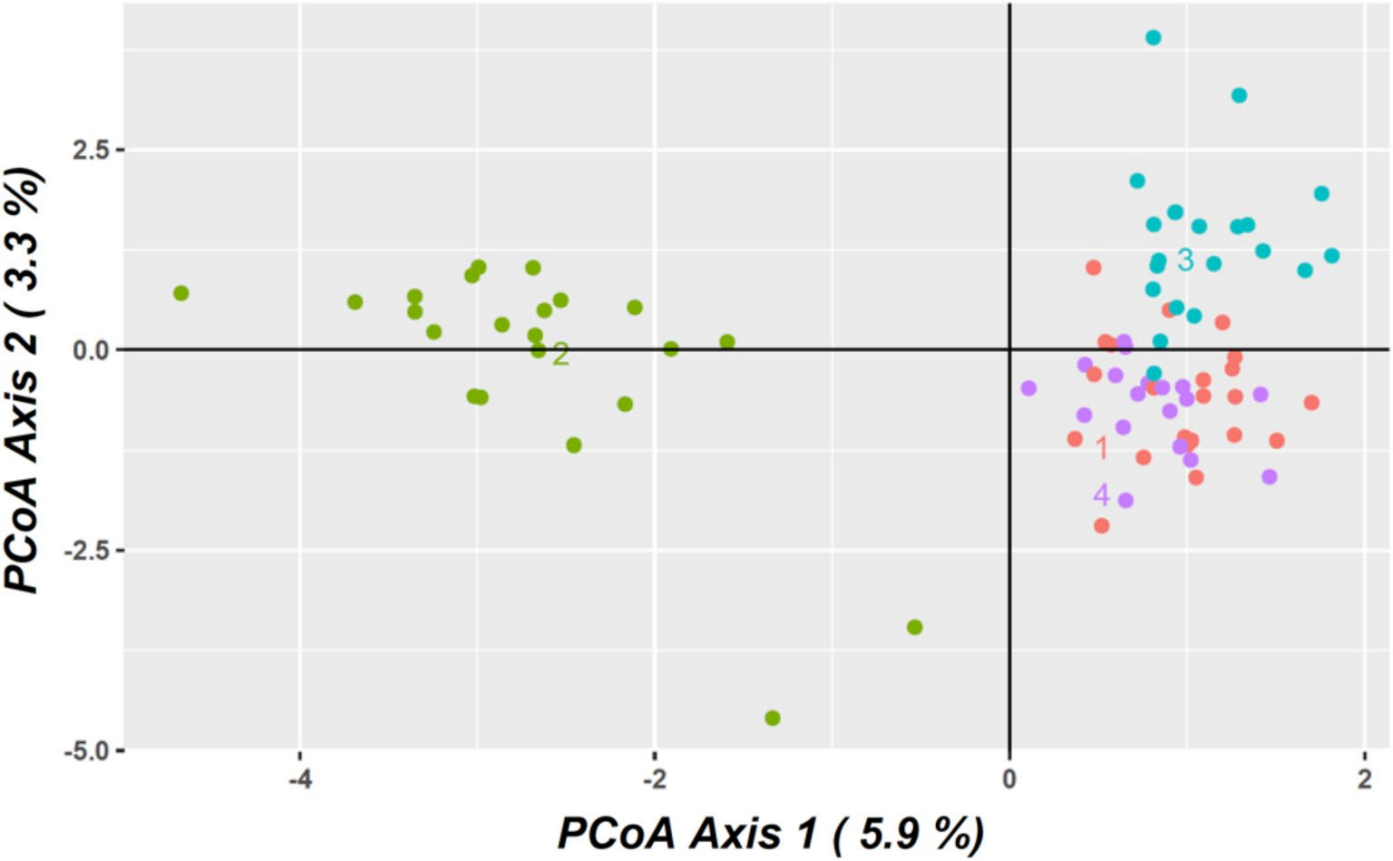
Principal Coordinate Analysis (PCoA) plot of the Lisbon arched-mouth nase *Iberochondrostoma olisiponense* samples performed on SNP genotypes. 1—Cabanas (red), 2—Trancão (green), 3—Muge (blue), 4—Tagus mainstem (purple).

The final dataset, including only *I. olisiponense* individuals, is composed of 83 genotypes, and 1,291 binary SNPs with 0.57% of missing data. Overall, genetic diversity indices were low across all sampled locations (Table 1), with the highest genetic diversity values found in Trancão and the lowest in Cabanas and Tagus main stem. The distribution of the genetic diversity in the PCoA (Fig. 3) shows that individuals from Trancão form a cluster apart from those of others locations along the PC1 axis, suggesting marked genetic divergence between downstream and upstream sites. In turn, samples from Muge form a cluster separate from those of the Tagus main stem and Cabanas along the PC2 axis, further suggesting genetic differentiation among the upstream sites. Moreover, the PCoA plot revealed the presence of two individuals from Trancão located between the two main groups of individuals along the PC1 axis.

**Table 1:** Genetic diversity estimates per sample location of the Lisbon arched-mouth nase *Iberochondrostoma olisiponense.*

Sample location	N	H_o_	H_e_	A_r_	*F*_IS_
Cabanas	22	0.11	0.12	1.5	0.076
Trancão	22	0.15	0.15	1.6	0.051
Muge	21	0.13	0.14	1.6	0.066
Tagus mainstem	18	0.12	0.13	1.6	0.053

N—sample size; H_o_—observed heterozygosity; H_e_—gene diversity; A_r_—allelic richness; *F*_IS_—inbreeding coefficient.

### Genetic differentiation and population structure

*F*_ST_ values between pairs of sample locations were low yet significant (*p* < 0.001), except between Cabanas and Tagus. The highest *F*_ST_ values were found in pairwise comparisons including Trancão, followed by those involving Muge (Table 2). These results are consistent with the largest number of private alleles being found in Trancão, followed by Muge (Supplemental Table 1). Conversely, the number of shared alleles was highest between Cabanas and Tagus (Supplemental Table 1).

**Table 2:** Pairwise *F*_ST_ (below the diagonal) and *P* values (above the diagonal) among Lisbon arched-mouth nase *Iberochondrostoma olisiponense* sample locations.

Sample location	Cabanas	Trancão	Muge	Tagus mainstem
Cabanas		< 0.001	< 0.001	0.067
Trancão	0.061		< 0.001	< 0.001
Muge	0.026	0.065		< 0.001
Tagus mainstem	0.003	0.054	0.024	

**Fig. 4:**
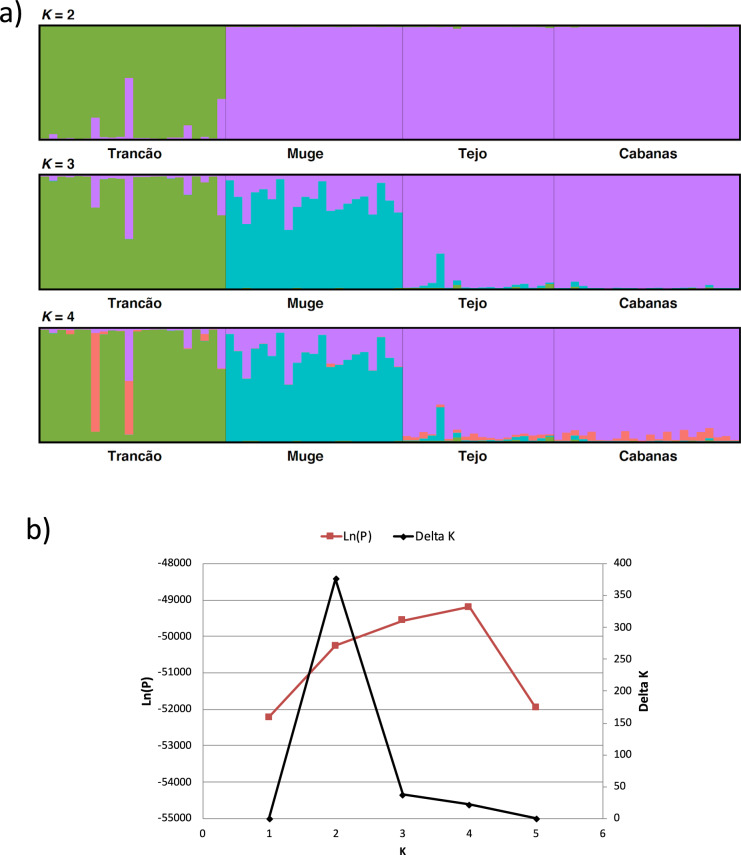
STRUCTURE analysis of the Lisbon arched-mouth nase *Iberochondrostoma olisiponense* samples. (**a**) Bar plot of the posterior probability of the coefficient of membership, each vertical line represents an individual and colours represent the inferred ancestry from *K* populations. Results for *K* = 2 to *K* = 4 are shown; (**b**) Plot of the Log posterior probability (Ln(P), red line) and Evanno’s method (Delta K, black line) vs *K.*

Structure detected *K* = 2 as the most probable number of clusters when using the Evanno’s method, whereas the standard method showed *K* = 4 having the highest likelihood values (Fig. 4). For all *K* values, Cabanas and Tagus individuals clustered together, whereas those from Muge and Trancão formed distinct clusters at K > 2. The coefficient of membership plots (Fig. 4) for K = 4 showed that two individuals from Trancão had marked admixed ancestries with predominant composition of a fourth cluster not present in high percentage in any of the sampled sites, suggestive of an unsampled source population. Interestingly, these individuals are concordant with those located away from the two main clusters found in the PCoA (Fig. 3). The presence of admixed ancestry from the Tagus/Cabanas cluster in all individuals from Muge and in some from Trancão (Fig. 4) suggests past genetic connectivity across the sampling area.

**Fig. 5:**
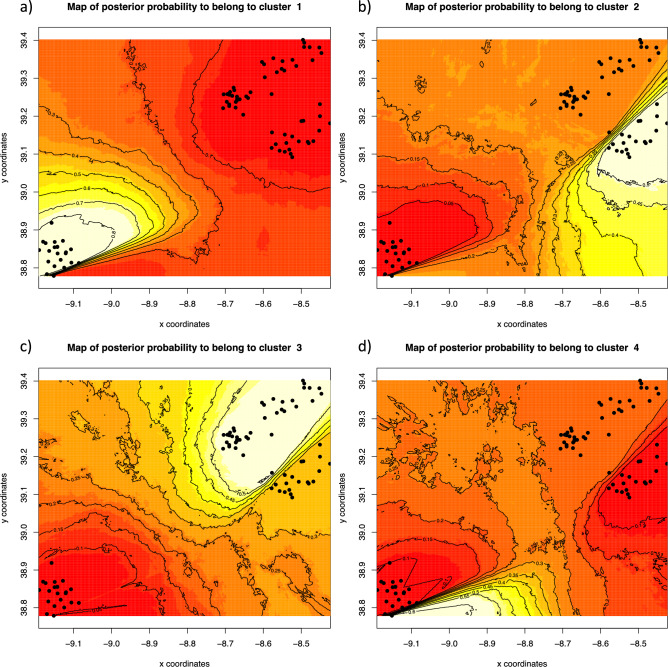
GENELAND results for *K* = 4 using the spatial model with correlated allele frequencies. Plots representing the assignment of pixels to the four clusters. The highest membership values are in light yellow and the contour lines indicate the spatial position of genetic discontinuities between populations. Cluster 1 includes all but one individual from trancão; Cluster 2 includes all individuals from Muge; Cluster 3 includes all individuals from Tagus mainstem and Cabanas; Cluster 4 includes a single individual from Trancão (lower left corner).

The spatially explicit model implemented in geneland with correlated allele frequencies reached convergence of MCMC and the highest likelihood value at *K* = 4 (Supplemental Figure S3). Individual assignments performed in geneland with *K* = 4 revealed that samples from Muge and Tagus/Cabanas were distributed in two separate clusters, while all individuals from Trancão except one comprised a third cluster (Fig. 5a–c). A fourth cluster included a single individual from Trancão (Fig. 5d), suggesting the presence of an unsampled population.

In the case of fineRADstructure (Supplemental Figure S4), individuals were clustered into three major groups corresponding to Trancão, Muge and Tagus/Cabanas. However, three individuals from Trancão shared high co-ancestry with two individuals from Tagus in the Tagus/Cabanas group, suggesting recent gene flow between these locations. Furthermore, some individuals in Trancão display high levels of co-ancestry, suggesting a high level of relatedness among them.

### Demographic analyses

**Fig. 6:**
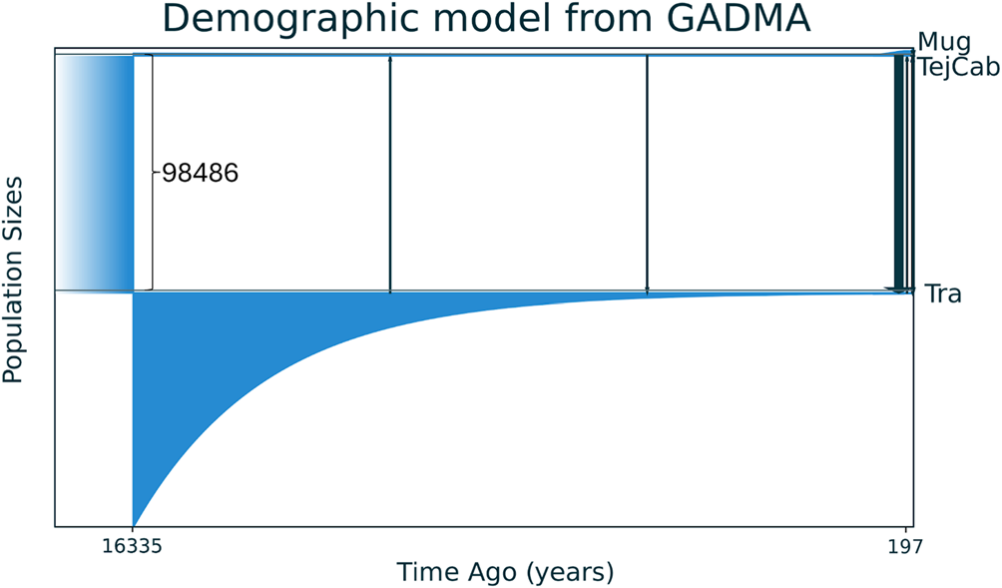
GADMA analysis result showing a first population split between Trancão and upstream locations (Muge/ Tagus/Cabanas), and a more recent split between Muge and Tagus/Cabanas. Times is in years before present. Effective population size is depicted in blue with thickness of the lines being proportional to size. Vertical grey arrows indicate events of migration between populations, with line thickness proportional to number of migrators, and direction of arrow indicating direction of gene flow.

No outlier SNP locus was detected using Bayescan, thus all SNP loci in the final dataset were used in the demographic analyses. The population effective size results showed reliable confidence intervals except for the Muge sample when using the jackknife method, where an infinite upper interval was obtained. The lowest *N*_*e*_ value was detected in Trancão, although in general all the samples showed low *N*_*e*_ estimates ranging from ~ 100 for Tagus/Cabanas and Muge to ~ 30 for Trancão (Table 3). All estimates had similar values when using different values of *Pcrit*, suggesting that minor allele frequencies have little influence on the *N*_*e*_ estimates. Historical demographic reconstructions based on gadma highlighted a split between the Trancão and Tagus populations at about 16,000 years ago (Fig. 6). While the obtained divergence time may not be precise, since the mutation rate used was estimated for other taxa, we do not expect it to differ by orders of magnitude and the divergence time should fall within a similar time scale to the one estimated here. The simulations indicate that the population at the time of split was centered in Trancão, followed by an exponential decrease in size until current levels, with some episodes of gene flow both upstream and downstream. The split between Muge and Tagus/Cabanas is estimated to have occurred in very recent historical times, after a period of a small increase in population size (Fig. 6).

**Table 3:** Estimates of effective population size (Ne) of the Lisbon arched-mouth nase *Iberochondrostoma olisiponense *populations using NeEstimator v2.

Population	N	P_crit_	Independent alleles	Ne	CIs for Ne			
					Parametric		Jackknife method	
Cabanas + Tagus mainstem	40	0.05	141504	107.6	99.2	115.0	68.2	216.5
		0.02	291903	98.7	93.2	102.5	65.4	176.3
		0.01	400746	99.7	94.7	102.7	70.5	155.8
Trancão	22	0.05	152648	35.2	33.2	37.1	15.7	355.0
		0.02	373722	25.6	24.6	26.0	19.1	35.6
		0.01	373722	25.6	24.6	26.0	19.1	35.6
Muge	21	0.05	123674	191.5	147.3	262.5	61.5	Infinite
		0.02	376479	77.9	73.7	85.1	45.7	232.9
		0.01	376479	77.9	73.7	85.1	45.7	232.9

Cabanas and Tagus mainstem samples were grouped together as their pairwise *F*_ST_ was not significantly different (details in Table 2). N—number of samples; P_crit_—minor allele frequency to be screened out; CIs for Ne—95% confidence intervals for the Ne estimates.

## Discussion

Our results show marked genetic population structure in the Lisbon arched-mouth nase coupled to small effective population sizes of current population nuclei. Genetic differentiation was detected among all tributaries: the strongest divergence was observed at the site located furthest downstream (i.e., Trancão) while lower but significant genetic differentiation was found between upstream locations (i.e., Muge and Cabanas/Tagus main stem). Individuals from the Cabanas tributary were genetically indistinguishable from those sampled in the Tagus main stem suggesting connectivity between these locations. Indeed, the hypothesis that the species may occur in Cabanas only intermittently has been suggested earlier based on the lack of recruits and on the presence of adults restricted to a small area immediately adjacent to the Tagus main stem^17^.

Our results further suggest the presence of additional unsampled population nuclei of the Lisbon arched-mouth nase. This was most evident in Trancão where some admixed individuals had genetic ancestry not found in the remaining sampled sites (Figs. 4 and 5). This may be due to the presence of additional populations undetected during our field survey or currently extinct. Indeed, historical records of species´ presence include sites located along the lower section of the sampled area^21^, of which some were not surveyed here^17^ (e.g., lower section of Rio Maior—Vala da Azambuja) due to recurrent and continuous pollution events in these sites^19^. The potential for additional population nuclei and a wider spatial distribution of the Lisbon arched-mouth nase is also supported by recent records of this species in the tidal oligohaline and mesohaline sections of the Tagus mainstem, ~ 40 to 50 km upstream from the confluence with Trancão^18,19^. These sections have regular occurrences of other freshwater fishes, such as the cyprinid *Luciobarbus bocagei* and the leuciscid *Pseudochondrostoma polylepis* (^22,23^and Costa J.L., pers. communication). Indeed, some relatives within the family Leuciscidae occurring in other large estuaries in similar environments exhibit tolerance to water salinities up to 18%o (e.g.,^24–27^), suggesting a similar tolerance for *I. olisiponense*.

While current genetic connectivity appears restricted among the population nuclei detected in the Lisbon arched-mouth nase, we found evidence of sporadic gene flow at historical and contemporary time scales. Specifically, pulses of past gene flow between the diverged populations were reconstructed by GADMA (Fig. 6), and admixed individuals were found in Trancão (Fig. 4, and Supplemental Figure S4). These observations suggest that there were periods of suitable hydrological conditions in the past that allowed for long- distance movement of individuals along the lower Tagus basin. Indeed, connectivity along the Tagus main stem may have occurred during sporadic periods of high fluvial activity (i.e., high freshwater inflow) recorded in the last 10 000 yr BP^28^. Presently, low salinity conditions (< 10 ppt) occur during winter and last about for up to 3 weeks at CoastNet Station located ~ 4 km downstream from the Trancão river mouth. This condition creates a wide oligohaline-mesohaline section in the upper Tagus estuary, which could allow some degree of connectivity between freshwater populations. Moreover, the CoastNet Station located ~ 11 km upstream of the Trancão outlet, has low salinity values (< 10 ppt) for more than 5 months per year (data from 2019 to 2023; http://geoportal.coastnet.pt/).

Since the estimated time of speciation of the Lisbon arched-mouth nase, ~ 12.5–7.9 Myr before present (BP) in the late Miocene^29^, the lower Tagus basin has undergone major reworkings of its morphology and environmental conditions. Notably, the region has alternated between freshwater and brackish/saltwater conditions due to marine regressions and transgressions during glacial and interglacial periods^30–33^. Changes in relative sea level and sediment supply in the last 30 kyr BP have shaped the lower Tagus basin from a deep incised channel within a broad fluvial plain during the lowstand period, to the development of extensive marsh areas and a broad estuarine–marine central basin upon rapid sea-level rise^34^. The effects of these shifts through time may vary among species, depending on their specific habitat requirements. For instance, the Lisbon arched-mouth nase is strongly associated with lowlands of riverine habitats with slow current, soft substrate and abundant riparian and aquatic vegetation, including seasonal wetlands^17^. Although salinity tolerance was never tested for the Lisbon arched-mouth nase, it is likely that it does not tolerate salinities larger than 15 ppt (see above for details on other leuscicids). Such habitat requirements may have resulted in habitat tracking during sea level fluctuations, as well as in recent habitat loss/contraction driven by human-mediated pressure in the Lisbon arched-mouth nase, shaping its current genetic diversity distribution.

The current pattern of genetic differentiation in the Lisbon arched-mouth nase may comply with expectations of isolation-by-distance, with more distant populations exhibiting large genetic distances. However, the timings of population divergence reconstructed here (Fig. 6) suggest that processes occurring at evolutionary time-scales may be the main drivers of the observed genetic structure. For instance, differentiation of the most downstream population (Trancão) from those further upstream appears to have occurred first, and to date far back into the past compared to the much more recent split of the two upstream population nuclei (Fig. 6). Within the geological context of the lower Tagus section, gene flow among tributaries via the Tagus main stem was likely restricted until current sea levels were reached (< 4 000 yr BP) concomitant with floodplain and levee development in the mid- and upper- basin sections^33,35^. Limited among-tributary connectivity was likely felt during low-stand periods, when the Tagus was then braided river system with a steep gradient fed by fast flowing water carrying an abundant supply of coarse sediments^33^ and thus unsuitable to the Lisbon arched-mouth nase. In turn, salt/brackish water conditions may have extended into the lower reaches of some of the Tagus River tributaries during high-stand periods, also limiting among-tributary connectivity (e.g., Muge^31,36,37^).

The recent divergence of upstream populations suggests more contemporary drivers of genetic discontinuity among tributaries. Water usage has grown dramatically in the last century in the lower Tagus section and major changes in river flow dynamics have occurred associated with increase damming, river regulation and water transfer^20,38,39^. Seasonal floods in the lower Tagus basin during winter have decreased dramatically in frequency and intensity since 1970, associated with human-driven changes in the river channels and, more recently, construction of several reservoirs^20,38,39^. In small tributaries, such as Muge, former wetlands have been drained for agricultural practices or transformed into rice paddies, and former river channels have been cleared of riparian vegetation with water flow controlled through a system of small levees and/or gates (Ribeiro F., Veríssimo A., pers. observations). Such pronounced habitat changes coupled to regulation of flow regimes may contribute to a recent decrease in gene flow between tributaries and/or the river main stem.

The low occupancy and restricted distribution of the Lisbon arched-mouth nase, which occurs exclusively on alluvial floodplain and tidal freshwater habitats, may be the result of a historically limited range of suitable habitats. These lowlands are characterized by sandy and mud substrate with slow current, and abundant aquatic vegetation with some riparian trees such as willows (*Salix* sp.)^10^. Contraction and expansion of these habitats associated with global climate shifts and sea level changes occurred repeatedly during an extended time period^33,35^, including the age of many contemporary taxa. Such conditions could have severely limited population growth and genetic diversity in inland wetland taxa occupying riverine lowland regions near estuaries. On the other hand, it may be expected that populations may have persisted mainly in areas upstream of the recurrent brackish/saltwater influence of marine transgressions. Thus, population nuclei of the Lisbon arched-mouth nase persisting in pockets of suitable habitats may have served as the source of recruits for new populations and of migrants connecting populations in different tributaries, such as Maior and Magos^13^, upon re-establishment of more broadly distributed suitable environmental conditions.

The consistently small effective population size of upstream populations of the Lisbon arched-mouth nase may reflect such historically variable but generally limited availability of suitable habitats, coupled with restricted or absent connectivity among tributaries. Such conditions may have limited population size increase and expansion into the mid-section of the lower Tagus basin over the last 16 000 yr BP due to a wide marine intrusion^5,35,40^. Favorable conditions re-established in the more recent past (~ 4000 yr BP) may have expanded the area of suitable Lisbon arched-mouth nase habitats to those observed nowadays. During this period, fluvial connectivity among tributaries may have been restored, potentially leading to a slight increase in population size in upstream locations (i.e., Muge and Tagus/Cabanas; Fig. 6). Despite this, the extent of suitable wetland habitats in the upper section of the lower Tagus basin may have been limited by a combination of human-driven land use changes (e.g. deforestation and land reclamation for agriculture and pasture), coupled to a decrease in rainfall particularly in the last 3000 y BP^5^. On the other hand, continuous contraction of suitable habitat in downstream populations (e.g., Trancão) may have promoted a continuous population size decrease. Indeed, the area occupied by floodplains in the lowermost section of Tagus basin has been decreasing since the past ~ 10,000 y BP, associated with the sea-level rise and invasion of saltwater into the estuary and of brackish waters into areas of previous freshwater floodplains^33^. Currently, the Trancão population nucleus is located in an area of high human occupation and intensive agricultural practices, with high water demand particularly during the dry season. This often leads to severe reductions of the local wetland area with aquatic species being confined to a few man-made wells that were previously used for irrigation and are naturalizing due to lack of maintenance (Fig. 1; Santos D., Gante H., Ribeiro F., Veríssimo A., pers. observations). These semi-natural wells have dirt walls, with 3–5 m deep and have a diameter of ~ 6 m, being encircled by giant cane (*Arundo donax*) and halophytic plant species (*Bolboschoenus* sp. and *Typha* sp.) (Santos D., Gante H., Ribeiro F., Veríssimo A., pers. observations).

The results shown here have important conservation implications. All population nuclei detected for the Lisbon arched-mouth nase showed low genetic diversity and correspondingly small effective population sizes (Tables 1 and 3), highlighting critical conservation concerns for the species. Small populations are more vulnerable to demographic, environmental, and genetic stochasticity compared to large populations, which elevates their extinction risk^41^. Also, low occupancy and population isolation reduce the probability of demographic rescue, which in turn impedes gene flow, increases genetic drift and ultimately heightens the risk of local extinction^42^. Habitat specialists occurring at low densities and with small geographical ranges display the highest risk^43^. This raises concerns regarding the adaptive potential and resilience of the Lisbon arched-mouth nase populations to future environmental changes. Indeed, future climate scenarios predict non-negligible impacts in rainfall regimes for the region; specifically, both longer dry seasons and more frequent droughts are expected due to rainfall reduction during spring and fall and, thus, to decreased annual precipitation (6% per decade till 2050)^44,45^. These more frequent and prolonged droughts could lead to local extinctions and/or strong population bottleneck effects due to wetland desiccation and habitat contraction/loss. It remains to be determined whether other inland wetland species inhabiting the lower Tagus exhibit similar vulnerability as reported in the Lisbon arched-mouth nase. However, such patterns may be expected for habitat specialist species of inland wetlands, which should be prioritized for research and conservation efforts.

While inland wetland communities may be adapted to the natural temporal variability of their ecosystems, this is not necessarily true for the most recent past where the pace and mode of variability are not natural but human-driven^7,11,12^. In the last century, the Lisbon arched-mouth nase has likely contracted both its distribution and abundance, with some populations seemingly driven to extinction in recent decades^17,21^. Indeed, the lower Tagus drainage has been subjected to an increasing set of human-induced pressures, such as land reclamation for urban and agricultural uses, pollution events, invasive species, and water extraction for human and agriculture consumption^19,20,46^. Such human-driven pressures are typical of inland wetland ecosystems^2,11,47^, underscoring the urgent need to understand the genetic diversity, population structure and connectivity of existing populations of lowland and tidal freshwater species to inform conservation prioritization and planning. Of particular urgency is more comprehensive inventory and characterization of inland wetlands and their associated biodiversity. For instance, about 30 inland wetlands were identified in the lower Tagus section recently for which there are no data on the resident fish community^19^. These efforts may uncover yet unknown population nuclei of species of conservation concern, such as the Lisbon arched-mouth nase.

In summary, we show genetic differentiation among small population nuclei in an endangered fish species with an exclusive distribution on alluvial floodplains and tidal freshwaters. Historical demographic reconstructions suggest that population isolation is consistent with periods of unsuitable habitat conditions while small population sizes may be due to limited available habitat coupled to restricted connectivity among tributaries. These conditions may result from the effect of marine transgressions and regressions on hydrological flow regimes and salinity, and more recently from human intervention on the landscape. This study may serve as a reference for taxa in similar inland wetland ecosystems, and may inform conservation efforts and planning given the predicted trends of population demographics, distribution and connectivity.

## Methods

### Sampling

The original sampling survey was conducted throughout the lower Tagus River basin for a total of 86 sites, including the main river stem and 19 tributaries, using electrofishing^11^. This study and its experimental protocols were approved by the Portuguese nature conservation authority (Instituto da Conservação da Natureza e das Florestas) under permit number 410/2016/CAPT, and were carried out according to relevant guidelines and regulations. Sites with reported species´ presence (including historical records) were largely confined within lowland wetland areas (mostly < 30 m in altitude^17^; Fig. 2). No large barriers (e.g., dams or weirs) are present along the sampling area, besides some century-old water mills of small height (generally less than 1 m high). The Lisbon arched-mouth nase was sampled from six sites (Fig. 2) distributed across three tributaries and the Tagus main stem: Cabanas (one site, n = 22), Muge (two sites, n = 25) and Trancão (one site, n = 22), and the Tagus main stem (two sites, n = 18). Two Portuguese arched-mouth nase individuals, *Iberochondrostoma lusitanicum*, originating from the Muge river were also genotyped to assess putative introgression with the Lisbon arched-mouth nase, detected in previous surveys^48^. Fish were anesthetized with MS222 and a small clip (~ 0.5 cm^2^) of the pelvic fin or lower caudal lobe was sampled and stored in 96% ethanol. Upon sampling, fish were allowed to recover from anesthesia before being returned live to their original location. This study is reported in accordance with ARRIVE guidelines.

### Data collection and laboratory procedures

Genomic DNA (gDNA) was extracted using a high-salt protocol modified for low yield samples following^49^. The final elution of gDNA was performed in 25 µl of nuclease-free water. The gDNA extractions were checked for quality by agarose gel electrophoresis, and quantified using Qubit (Invitrogen). Individual gDNA extracts were standardized to 400 ng in 20 µl and shipped to Diversity Arrays Technology (University of Canberra, Australia) for sequencing a reduced representation of the genome from double-digested restriction fragments using a DArTseq protocol. The resulting library was sequenced in an Illumina HiSeq 2500 as single-end reads of 77 nucleotides (nt), and genotyped using the proprietary DArTSoft14 pipeline. Around 30% of the samples were run as internal replicates to provide confidence levels in genotype calls.

### Data analysis

Only loci with reproducibility higher than 99% were retained. Moreover, additional SNP quality control filters were applied to retain loci with < 5% missing data, keeping individuals with > 80% of SNP loci genotyped, and keeping loci with Minor Allele Frequencies (MAF) > 1%. The presence of private alleles between sites was assessed with the function *gl.report.pa* of the *dartR 1.1.11* R package^50^. No monomorphic loci were detected after filtering. A global Hardy–Weinberg Equilibrium (HWE) and Linkage disequilibrium (LD) test was performed by GENEPOP 4.0^51^ using 10,000 dememorization steps of Markov chains, 20 batches and 5,000 iterations per batch. Loci not conforming to HWE expectations were excluded from further analyses.

Exploratory data analysis on the final filtered dataset aimed at assessing overall structure among multilocus genotypes, as well as identifying putative hybrid individuals. This was performed by Principal Coordinate Analysis (PCoA) and by constructing a neighbor-joining tree using *dartR 1.1.11* R package^50^. Genetic diversity indices, namely observed heterozygosity (Ho), gene diversity (He), allelic richness (Ar), and inbreeding coefficient (FIS) were calculated using the *hierfstat* R package 1.2.2^52^. Input datafiles for the different analyses were obtained by conversion of the original genlight object with the multilocus genotypes into a Structure format file (with the function *gl2structure* of the *dartR* package), and then converted into various formats using PGDSpider v2.0.6.0^53^.

Genetic differentiation was evaluated by means of pairwise *F*_ST_ values among sample collections, computed with the function *gl.fst.pop* of the *dartR 1.1.11* R package^50^ and 1000 bootstraps across loci to generate confidence intervals and p-values. STRUCTURE v2.3.4^54^ was run to assess overall population structure patterns based on inferences on the most likely number of K clusters (i.e., the probability of the data given *K* genetic clusters of individuals), and to estimate the probability of membership of each individual to each cluster using a Markov Chain Monte Carlo (MCMC) method. Structure 2.3 was run using sampling location information^55^ to improve inferences when there are small genetic differences between sample collections (as was the case here). We ran ten independent replicates for each value of *K*, ranging 1–5, using the admixture model with correlated allele frequencies^54,56^. Each run consisted of 100,000 MCMC steps with a burn-in period of 10,000 steps. For these analyses we used an approach described by^55^: the lambda value has been estimated by running the Structure 2.3 software for K = 2–4 allowing updates for lambda value. The optimum lambda value obtained was used to perform longer MCMC runs as described above. This strategy allowed modeling the frequency spectrum of SNPs which may be skewed towards rare alleles. The convergence of the MCMC runs was evaluated considering the extent of variation of the log probability among runs. The most likely number of clusters was inferred from both the standard method (i.e., plotting ln Pr (X|*K*) vs K) and the Delta *K* statistic^57^ based on a rate of change in the log probability of the data over consecutive *K* values. The results were averaged over multiple runs using the clummp software^58^ and plotted using the distruct program^59^.

Additionally, the spatially explicit Bayesian clustering program geneland 3.2.4^60^ was used to further investigate genetic structure and areas of genetic discontinuity. geneland considers individual multi-locus genotype data searching for the best fit to HWE and linkage disequilibrium, but also incorporates spatial data (i.e., individual geographical coordinates) to test the assumption that populations are spatially organized. geneland was run assuming a correlated allele frequency model with spatial information, thereby accounting for putative spatial correlation of genotypes. Any genetic boundaries detected are assumed to separate *K* random mating subpopulations, subdividing the space similarly to the Poisson-Voronoi tessellation^60,61^. Ten replicate runs were performed for each *K* value, ranging 1–5, using 500,000 MCMC steps, a thinning interval of 500 generations, a maximum rate of Poisson process fixed to 400, and spatial coordinates uncertainty of 2.5 km. The prior for allele frequencies was modeled assuming a Dirichlet distribution^62^. The posterior probability of subpopulation membership was computed for each pixel of the spatial domain (400 × 400 pixels), using a burn-in of 500 iterations.

The fineRADstructure package v0.2^63^ was used with default settings to quantify the ancestry sources in each population. FineRADstructure utilizes a fineSTRUCTURE MCMC clustering algorithm^64^ to infer a co-ancestry matrix, which is a summary of nearest neighbor haplotype relationships across the dataset^63^.

Prior to demographic analyses, non-neutral loci were screened using Bayescan version 2.1^65–67^ with following run parameters: -n 5000 -thin 10 -nbp 20 -pilot 5000 -burn 50,000 -pr_odds 100. MCMC chain convergence and effective sample size were checked using the functions in the R package coda^68^, and putative outliers were plotted and assessed using the built-in R function *plot_bayescan*. Demographic inferences on the size of population nuclei of the Lisbon arched-mouth nase were made by calculating the contemporary effective population size based on the LD method (NeLD) using NeEstimator v.2.01^69^. Among several demographic and genetic methods available to estimate temporal and contemporary Ne, the linkage disequilibrium (LD) method^70^ performs best, even for small population sizes^71^. This method was run assuming different values of *Pcrit* to assess the impact of minor allele frequencies on the *N*_*e*_ estimates obtained.

Furthermore, GADMA^72^ was used to model the demographic history of the sampled populations. Like other demographic inference methods, GADMA is based on the comparison of allele frequency spectra (ASF) between a hypothetical model and the data. The difference being that GADMA automatically searches for the best model, in an unsupervised way, being only necessary to define a set of parameter constraints. Such a model assumes two sequential population splits in which we define a maximum complexity of two moments of demographic change before and after the second split and one moment before the first split. These included parameters such as population size and its variation, segregation time, and migration rates. Timings of demographic change were estimated based on a mutation rate estimated for fish ddRAD loci of 6.6 × 10^–8^ per site per generation^73^, and a generation time of 2 years. SNPs were considered to be unlinked. Model evaluation was performed with Moments^74^ using a down projected ASF dimensions (Trancao: 25, Tagus/Cabanas: 46, Muge: 27), which according to a test performed with easySFS (https://github.com/isaacovercast/easySFS) keeps at least 90% of the segregation sites. We performed three independent searchers using the local optimizer powell and the best model was chosen based on Akaike’s information criterion.

## Supplementary Information

Supplementary Information.

## Data Availability

Genotypes called for the 83 individuals for the Lisbon arched-mouth nase *Iberochondrostoma olisiponense* used in the analyses are available for download as file “Iolisiponensis_SNPgenotypes_genepop.txt” in genepop format at the link https://data.mendeley.com/preview/r4b5mdzjt9?a=d696afc8-5d2a-4e1c-962f-185d851977d7. Raw read sequences were deposited in GenBank under accession no. PRJNA1207780.
