# Supplementary material for: Genetic structure and historical demography of inland wetland fish using the endangered Lisbon arched-mouth nase as a case-study
Source: Sci Rep. 2025 Jul 2;15:22998. doi: 10.1038/s41598-025-05280-x (PMC12215544; doi:10.1038/s41598-025-05280-x)
Supplement: Supplementary file 1 — Supplementary Information. [file 41598_2025_5280_MOESM1_ESM.pdf]

Supplemental File

Understanding the genetic patterns of inland wetland fish: the endangered Lisbon arched-mouth nase as a case-study

Authors: Giulia Riccioni[1], Manuel Curto[2,3,4], Carlos D. Santos[5], Maria Judite Alves[6,7], Hugo F. Gante[7,8,9], Filipe Ribeiro[2], Ana Veríssimo[3,4]*

Supplemental Table 1. Private alleles per pair of sample locations. N1 – no. of samples in location 1; N2 – no. of samples in location 2; mdf=mean allele frequency difference between loci. Tagus – Tagus main stem.

Location 1	Location 2	N1	N2	fixed	Private in location 1	Private in location 2	Total private alleles	mdf
Cabanas	Trancão	22	22	0	219	335	554	0.075
Cabanas	Muge	22	21	0	163	282	445	0.055
Cabanas	Tagus	22	18	0	129	146	275	0.038
Trancão	Muge	22	21	0	306	309	615	0.081
Trancão	Tagus	22	18	0	313	214	527	0.073
Muge	Tagus	21	18	0	283	181	464	0.056

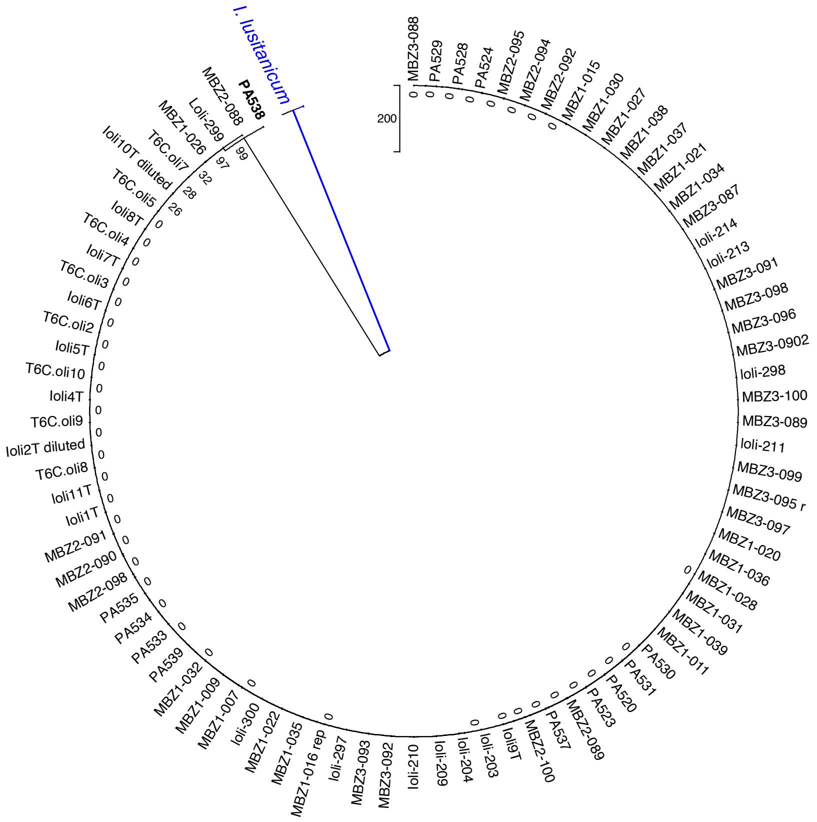

Figure S1. Neighbor-joining tree including both *Iberochondrostoma olisiponense* (black font) and *I. lusitanicum* (blue font). The tree shows one hypothetical hybrid individual (highlighted in bold), morphologically identified as *I. olisiponense*, clustering as basal to the remaining *I. olisiponense* samples.

a)

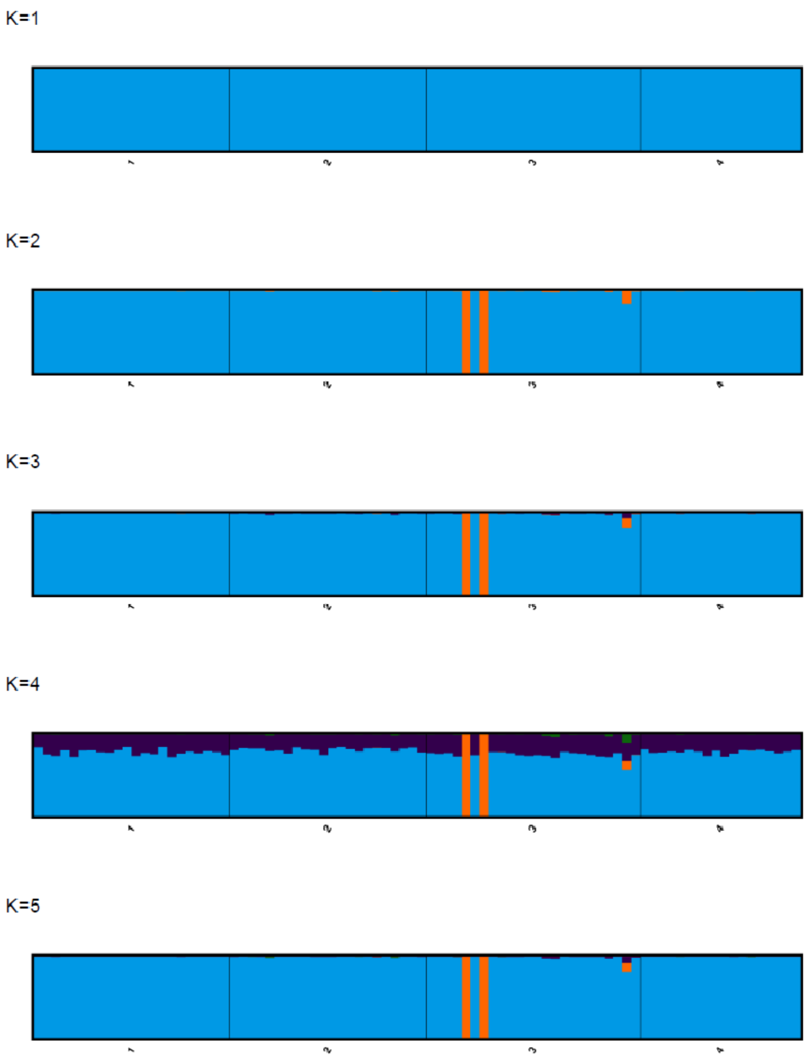

b)

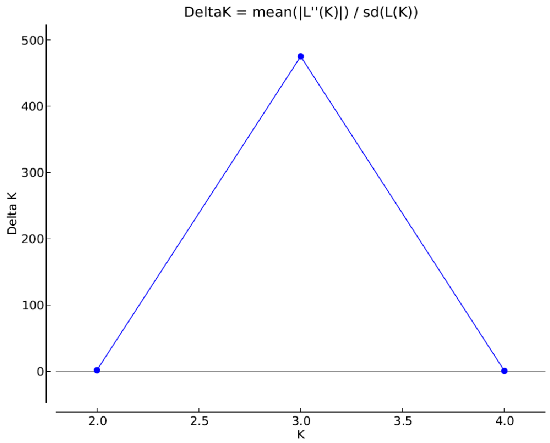

c)

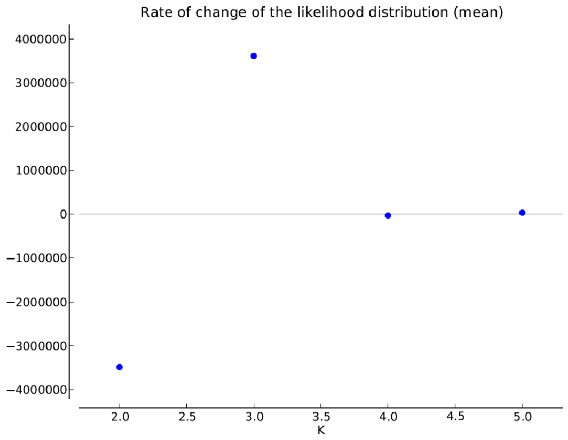

Figure S2. STRUCTURE analysis for the data set including both *Iberochondrostoma olisiponense* and *I. lusitanicum*. a) Bar plot of the posterior probability of the coefficient of membership, results are shown for K = 1–5; b) Evanno's method plot; c) Plot of the Log posterior probability vs K.

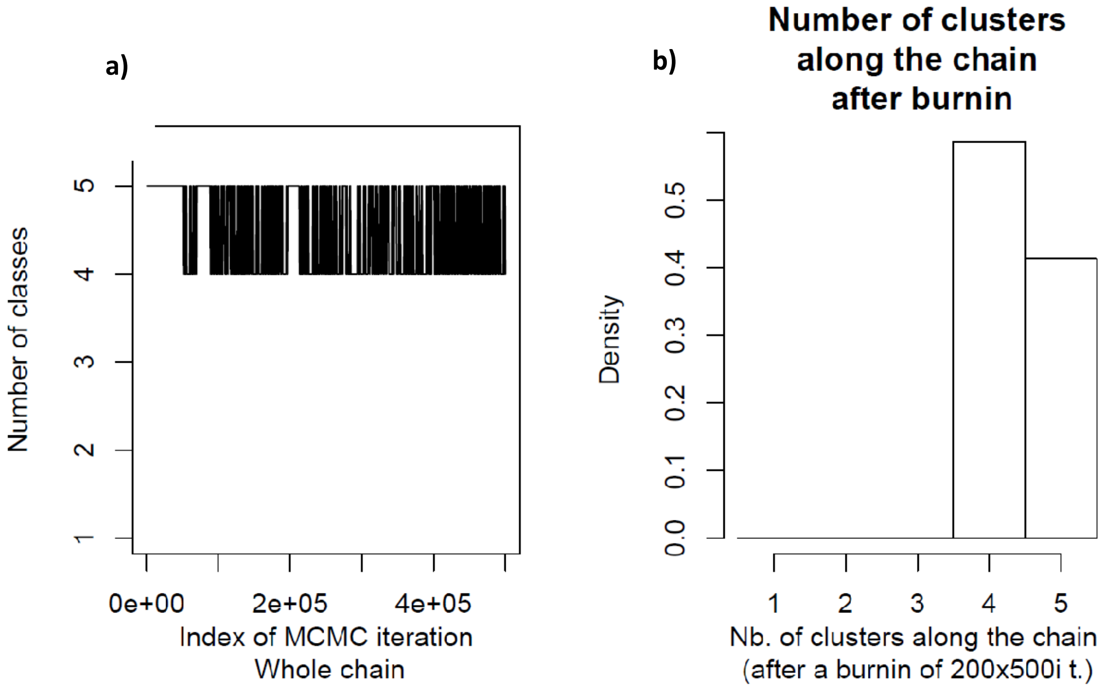

Figure S3. GENELAND results of *Iberochondrostoma olisiponense* for *K* = 4. a) Index of MCMC iteration plot, and b) number of clusters after burn-in.

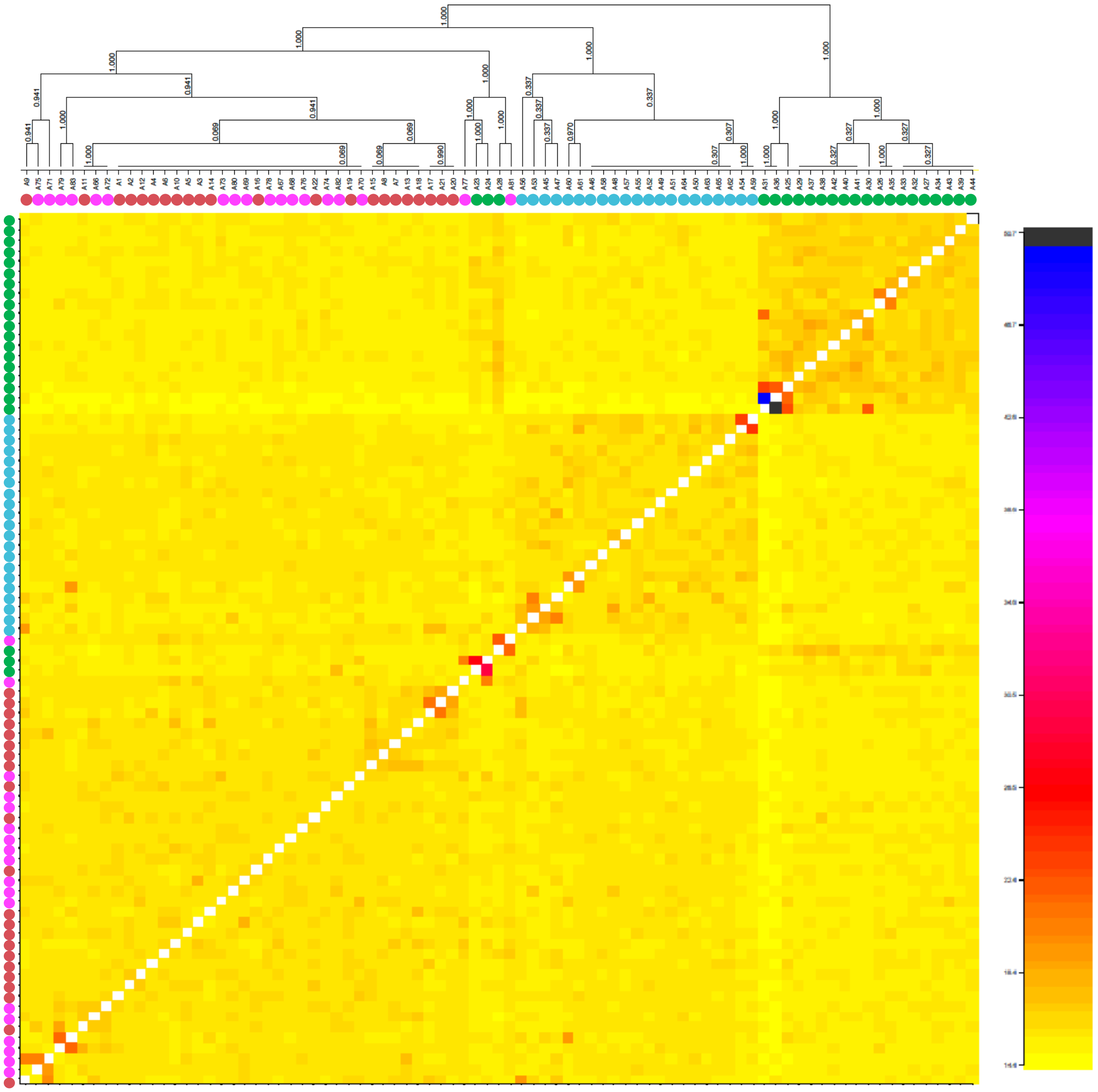

Figura S4. FineRADstructure coancestry map and tree of *Iberochondrostoma olisiponense* individuals. The right-hand side panel indicates the coancestry levels with light yellow indicating low coancestry, and darker yellows, oranges and reds indicating progressively higher coancestry. Individuals are color labelled according to sample location: green – Trancão; blue – Muge; red – Cabanas; pink – Tagus main stem.
